# Study on Stability of Remifentanil, Sufentanil, and Their Metabolites in Human Whole Blood and Urine

**DOI:** 10.3390/metabo15120804

**Published:** 2025-12-18

**Authors:** Zhuoyi Wang, Huan Gao, Yingwen Xu, Di Liang, Xian Ju, Kaili Du, Xiaoxi Mu, Xi Zhang, Ziyang Dong, Tao Wang, Dan Zhang, Zhiwen Wei, Jianguo Li, Keming Yun, Zhe Chen

**Affiliations:** 1 School of Forensic Medicine, Shanxi Medical University, Jinzhong 030600, China; 2 Shanxi Key Laboratory of Forensic Medicine, Jinzhong 030600, China; 3 Key Laboratory of Forensic Toxicology of Ministry of Public, Jinzhong 030600, China; 4 Department of Pathology, Shanxi Medical University, Taiyuan 030001, China; 5 Taiyuan Railway Public Security Bureau Linfen Public Security Department, Linfen 041000, China; 6 China Institute for Radiation Protection, Taiyuan 030001, China

**Keywords:** stability, remifentanil, sufentanil, metabolites, HPLC-MS/MS

## Abstract

Background: The accurate detection of remifentanil and sufentanil in biological samples is challenged by their rapid metabolism and instability, complicating clinical and forensic toxicology analysis. This study aimed to evaluate the stability of remifentanil, sufentanil, and their primary metabolites—remifentanil acid and norsufentanil—in human whole blood and urine. Methods: A high-performance liquid chromatography–tandem mass spectrometry (HPLC-MS/MS) method was developed and validated for simultaneous quantification, demonstrating satisfactory linearity (0.10–200 ng/mL, r^2^ > 0.99), detection limits (0.01–0.20 ng/mL), and recovery rates (85.06–119.42%). Stability was assessed under varying temperatures (4 °C, −20 °C, −80 °C) and anticoagulant conditions (EDTA-K_2_, sodium heparin, sodium citrate) over 35 days. Results: Remifentanil exhibited significant instability in whole blood, degrading over 50% within 6 h at 4 °C, whereas stability was markedly improved at −80 °C and in sodium citrate-containing samples. Remifentanil acid remained stable for up to 35 days at −80 °C. Sufentanil was generally more stable, particularly at −80 °C in both blood and urine, while norsufentanil remained stable for 7 days at −20 °C in citrate-anticoagulated blood but degraded rapidly at 4 °C. These findings support specific recommendations for sample preservation, including storage at −80 °C and the use of sodium citrate as an anticoagulant, to enhance detection reliability in toxicological and pharmacokinetic studies.

## 1. Introduction

Remifentanil and sufentanil are synthetic opioid analgesics used as anesthetics for short-duration surgical procedures. However, as fentanyl analogs, remifentanil and sufentanil exhibit great potential for abuse [1]. Fatalities associated with fentanyl analogs, including remifentanil and sufentanil, have increased due to the rise in clandestine use over the past decade, posing a significant threat to global public health and safety [2,3]. Thus, accurate detection of remifentanil and sufentanil is critical for clinical drug monitoring and the identification of illicit drugs in forensic toxicology.

**Figure 1:**
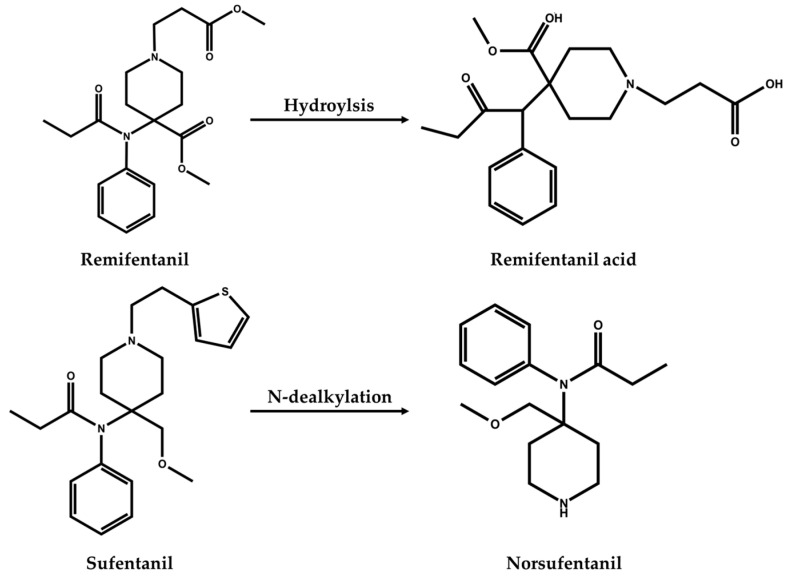
Chemical structures of remifentanil, sufentanil, and their metabolites.

Remifentanil and sufentanil, like fentanyl, exhibit high potency and undergo rapid and extensive metabolism, which makes the detection of the fentanyl analogs in biological matrices challenging. As shown in Figure 1, remifentanil is predominantly metabolized via ester hydrolysis catalyzed by organ-independent non-specific esterases, resulting in the formation of its inactive metabolite, remifentanil acid [4]. Remifentanil was reported to have a half-life of approximately 3 min [5], which contributed to its inherent instability. Moreover, the presence of an N-substituted methyl propanoate ester group that is highly susceptible to degradation by endogenous esterases and chemical hydrolysis [6], thus after blood sampling, remifentanil remains unstable in whole blood and plasma. In contrast, sufentanil undergoes primary metabolism in the liver via the cytochrome P450 enzyme system, where N-dealkylation generates norsufentanil, a metabolite that retains significant pharmacological activity (Figure 1) [7]. Based on the above metabolic characteristics of remifentanil and sufentanil, it is therefore increasingly important to understand the stability of drug concentrations in biological samples during storage to ensure accurate clinical drug monitoring, as well as pharmacokinetic studies.

To date, most studies have primarily focused on developing highly sensitive analytical methods for detecting fentanyl analogs; however, few have examined the stability of remifentanil and sufentanil in human biological samples. Recently, Jung et al. found that remifentanil was stable in whole blood samples stored at −20 °C but degraded by more than 50% after just 1 day at 4 °C, indicating significant instability [8]. Similarly, Koster R.A et al. reported a 20% decrease of remifentanil in whole blood stored at 4 °C for 5 h [9]. Moreover, they demonstrated that matrix acidification enhances remifentanil stability: the addition of formic acid to EDTA-K_2_ (ethylenediaminetetraacetic acid)-preserved blood increased remifentanil stability at 4 °C from 5 h to 14 days. Regarding the stability of sufentanil, as early as 2001, it was reported that sufentanil in plasma stored at 4 °C in nonsilanized tubes began to degrade within the first hour [10]. Additionally, significant degradation was observed after 8 h of storage at −25 °C in nonsilanized glass tubes. Even in silanized glass tubes, sufentanil stability at −25 °C was limited to only 8 h. Additionally, Andreas et al. investigated the long-term stability of sufentanil and found that sufentanil concentrations in human plasma samples stored at −20 °C for over 11 years decreased by approximately 60% [11]. Most stability studies on fentanyl analogs have focused on factors such as sample pH, storage temperature, and container material; however, the stability of metabolites, particularly in the context of forensic toxicological analysis, has received relatively little attention. Drug metabolites generally exhibit extended detection windows compared to their parent compounds, making metabolite analysis an effective strategy to minimize false-negative results associated with sole detection of the parent drugs. Consequently, characterizing metabolite stability provides valuable forensic guidance for accurate identification of drug abuse, including fentanyl analogs and other illicit controlled substances [12,13].

Unlike most studies that have investigated stability through forced degradation experiments [14,15,16], this study aims to systematically evaluate the stability of remifentanil, sufentanil, and their primary metabolites (remifentanil acid and norsufentanil) in human whole blood and urine under real sample storage conditions. Specifically, two critical factors are examined: (1) storage temperature, including refrigerated at 4 °C, frozen at −20 °C, and ultra-low at −80 °C; (2) the type of anticoagulants used in vacuum blood collection tubes (EDTA-K_2_, sodium heparin, and sodium citrate). In this study, a high-performance liquid chromatography–tandem mass spectrometry (HPLC–MS/MS) method was developed for the simultaneous quantification of remifentanil, sufentanil, and their metabolites. This study underscores the critical importance of metabolite stability for ensuring reliable interpretation of pharmacokinetic data and forensic toxicological results, and provides a valuable reference for the optimal preservation of fentanyl analogs in biological matrices.

## 2. Materials and Methods

### 2.1. Chemicals and Reagents

Standard solutions of remifentanil (1 mg/mL), sufentanil (1 mg/mL), and norsufentanil (1 mg/mL) were purchased from Putian Tongchuang Technology Co., Ltd. (Shenzhen, China). Remifentanil acid was obtained from Shanghai Institute of Criminal Science and Technology (Shanghai, China). Fentanyl-d_5_ (internal standard, IS, 1 mg/mL) was purchased from Wendu Scientific Instrument Co., Ltd. (Guangzhou, China). Single-use negative pressure venipuncture tubes containing 1.00% sodium heparin, 1.50 mg/mL EDTA-K_2_, or 0.25% sodium citrate were purchased from Aosaite Medical Equipment Co., Ltd. (Shandong, China). Ammonium formate and citric acid were purchased from Shanghai Maclin Biochemical Technology Co., Ltd. (Shanghai, China). Formic acid, methanol, and acetonitrile of chromatographic grade were all obtained from Sigma-Aldrich (Shanghai, China).

### 2.2. High-Performance Liquid Chromatography–Tandem Mass Spectrometry Analysis

All the components were separated on a C18 column (Agilent Poroshell 120 EC-C18 2.1 × 100 mm, 1.9 μm) through an Agilent 1260 HPLC system (Agilent, Santa Clara, CA, USA). The injection volume was 5 μL. The mobile phase was mixed with solvent A (acetonitrile) and solvent B (ammonium formate, 20 mM. The column was held at 25 °C and eluted for 7 min in total at a flow rate of 0.20 mL/min with a gradient of 25% A (0–2 min), 85% A (2–2.5 min), 85% A (2.5–3.0 min), 25% B (3.0–3.5 min) and 25% A (3.5–7.0 min).

Quantification was performed in the multiple reaction monitoring (MRM) acquisition mode. Nitrogen was utilized as a nebulizer, drying, and desolvation gas. ESI in positive mode was used with a capillary voltage of 3500 V, a drying flow rate of 5.0 L/min, a nebulizer pressure of 45 psi, and a drying gas temperature of 300 °C. The other optimum MRM parameters for each analyte are shown in Table 1.

### 2.3. Sample Preparation

An aliquot of 200 μL per blood or urine sample was transferred to a 1.5 mL centrifuge tube, then the sample was mixed with 5 µL fentanyl-d_5_ (IS, 400.00 ng/mL). Next, 50% citric acid was added to the above mixture to adjust the pH to 4–5. The sample was then subjected to protein precipitation using acetonitrile (0.8 mL) and centrifuged for 10 min (13,000 rpm/min). The supernatant was taken into a 2 mL centrifuge tube and dried with a nitrogen stream, and the residue was redissolved with 100 μL mobile phase (acetonitrile: water/*v*:*v* = 1:3). Finally, the extract was filtered through a 0.22 μm organic membrane filter and injected into 5 μL of the extract for LC-MS/MS analysis.

### 2.4. Methodology Validation

#### 2.4.1. Standard Curve and Detection Limit

To prove linearity, calibrators were prepared by spiking 200 μL of blank blood (or urine) with increasing concentrations of remifentanil, sufentanil, remifentanil acid, and norsufentanil at 0.10, 0.50, 1.00, 5.00, 10.00, 50.00, 100.00, and 200.00 ng/mL, respectively, along with an IS at 10.00 ng/mL. The linearity was tested by plotting a calibration curve. The calibration curve was derived by plotting the peak area ratio (*y*-axis) of the target substance to the IS versus the concentration (*x*-axis) of the corresponding target substance. The LOD was defined as the lowest concentration of the analyte spiked in the matrix, which could be detected with a signal-to-noise (S/N) ratio of at least 3 [17].

#### 2.4.2. Accuracy, Precision, Extraction Recovery Rate, and Matrix Effect

Intra-day and inter-day precision were determined by analyzing six replicates at each of three quality control (QC) concentrations on the same day and over three consecutive days, respectively, and were characterized by the relative standard deviation (RSD, %). The three QC concentrations were set as the low concentration (0.50 ng/mL), the medium concentration (20.00 ng/mL), and the high concentration (150.00 ng/mL). Additionally, the extraction recovery and matrix effect were investigated by comparing the average peak areas (A) under three different conditions: group 1, where a standard solution at a certain concentration was prepared; group 2, where the corresponding concentrations of standard substances were added after extraction of different matrices; and group 3, where standard substances at corresponding concentrations were added before extraction of different matrices. The matrix effect was calculated as A_2_/A_1_ × 100%, and the extraction recovery was calculated as A_3_/A_2_ × 100%.

### 2.5. Stability Studies

#### 2.5.1. Whole Blood Samples

A total of 750 mL of blood was collected from twenty healthy volunteers (10 males, 10 females) using single-use vacuum venipuncture tubes. For each anticoagulant type (sodium heparin, EDTA-K2, and sodium citrate), 250 mL of blood was added to tubes containing the anticoagulant, which was subsequently mixed and divided into five equal portions. Group 1 served as a blank control, while the remaining four groups were each spiked with 100.00 ng/mL of remifentanil, remifentanil acid, sufentanil, and norsufentanil, respectively. After thorough mixing, each sample was further subdivided into three equal aliquots and stored at −80 °C, −20 °C, and 4 °C. Samples were extracted at various storage times: 0 d (initial), 0.25 d, 0.5 d, 1 d, 2 d, 3 d, 5 d, 7 d, 15 d, 25 d, and 35 d (with 3 replicates at each time point no outliers were excluded). Subsequently, remifentanil, sufentanil, and their metabolites were qualitatively and quantitatively detected using the developed HPLC-MS/MS method.

#### 2.5.2. Urine Samples

A total of 150 mL of urine was collected from six healthy volunteers (3 males, 3 females) and evenly divided into five equal groups. One group served as a blank control, while the other four groups were each spiked with 100.0 ng/mL of remifentanil, remifentanil acid, sufentanil, and norsufentanil (all spiked samples were subjected to ultrasonic mixing). Each group was further subdivided into three equal aliquots and stored at −80 °C, −20 °C, and 4 °C, respectively. The extraction time points and detection method were the same as those in Section 2.5.1.

### 2.6. Statistics

Statistical analysis was performed using IBM SPSS Statistics version 27.0.1.0 software. Results were presented as mean ± (standard deviation) SD. A one-way analysis of variance (ANOVA) was conducted on the hematological and urinalysis datasets, with *p* < 0.05 indicating statistical significance.

### 2.7. Ethics

The study was approved by the Ethics Committee of Shanxi Medical University (2021SLL021) and was conducted in accordance with the Declaration of Helsinki. Written informed consent was obtained from each participant.

## 3. Results

### 3.1. Method Validation

In this study, a sensitive and reliable HPLC–MS/MS method was successfully developed and validated for the first time to simultaneously detect remifentanil, sufentanil, and their major metabolites, remifentanil acid and norsufentanil, in whole blood and urine specimens. We implemented an optimized protein precipitation protocol using acetonitrile, with fentanyl-d_5_ as the internal standard (IS). Representative chromatograms of whole blood and urine samples spiked with each analyte were shown in Figures S1–S8. The retention times (Rt) of remifentanil, sufentanil, remifentanil acid, and norsufentanil in blood were 3.80 min, 1.96 min, 4.33 min, and 3.04 min, in urine were 3.79 min, 1.98 min, 4.33 min, and 3.02 min.

The validation data for the determination of remifentanil, sufentanil, and their metabolites in whole blood and urine samples were summarized in Tables S1 and S2. As shown in Table S1, the calibration curves for remifentanil, sufentanil, and their metabolites exhibited excellent linearity over the concentration range of 0.10–200.00 ng/mL, with correlation coefficients (r^2^) greater than 0.99. Furthermore, the limits of detection (LODs) of the method in whole blood were determined to be 0.06 ng/mL for remifentanil, 0.20 ng/mL for remifentanil acid, 0.03 ng/mL for sufentanil, and 0.02 ng/mL for norsufentanil, while the corresponding LODs in urine were 0.02 ng/mL, 0.04 ng/mL, 0.01 ng/mL, and 0.02 ng/mL, respectively. The extraction recovery for all analytes was satisfactory, ranging from 92.6% to 119.4% across the concentration range of 0.50 ng/mL to 150.00 ng/mL in both whole blood and urine (Table S2), demonstrating the efficiency of the protein precipitation-based sample preparation method. For all analytes, the matrix effect ranged from 88.4% to 106.9% in whole blood and from 85.06% to 113.4% in urine. The intra-assay and inter-assay accuracies for all analytes ranged from 93.45% to 115.21%, with corresponding precisions (RSD) ranging from 0.23% to 13.57%, all of which met the acceptance criteria of 85–115% for accuracy and ≤15% RSD for precision [18]. These results comply with the acceptance criteria (<±25% deviation) established by the German Society of Toxicology and Forensic Chemistry for LC-MS/MS analysis of drug of abuse in biological matrices, confirming the robustness of the analytical method across different biological matrices [19].

### 3.2. Stability Studies

#### 3.2.1. Stability of Remifentanil and Its Metabolite Remifentanil Acid

**Figure 2:**
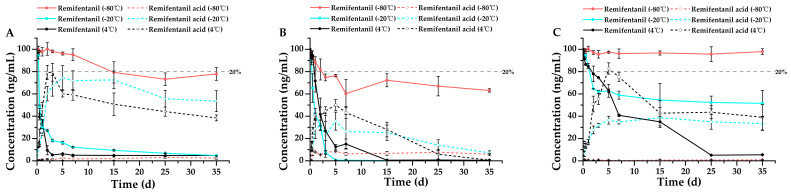
Stability profiles of remifentanil in whole blood stored at three temperatures (4 °C, −20 °C, and −80 °C) using three anticoagulants: (**A**) EDTA-K_2_ (*n* = 3, mean ± SD), (**B**) sodium heparin (*n* = 3, mean ± SD), and (**C**) sodium citrate (*n* = 3, mean ± SD). Detailed experimental data on the stability profiles during the first 3 days were presented in Figure S9 of the Supplementary Materials.

**Figure 3:**
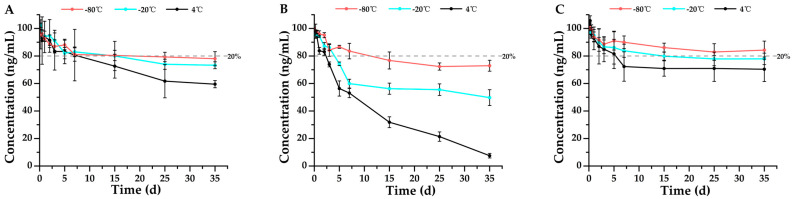
Stability profiles of remifentanil acid in whole blood stored at three temperatures (4 °C, −20 °C, and −80 °C) using three anticoagulants: (**A**) EDTA-K_2_ (*n* = 3, mean ± SD), (**B**) sodium heparin (*n* = 3, mean ± SD), and (**C**) sodium citrate (*n* = 3, mean ± SD). Detailed experimental data on the stability profiles during the first 3 days were presented in Figure S10 of the Supplementary Materials.

**Figure 4:**
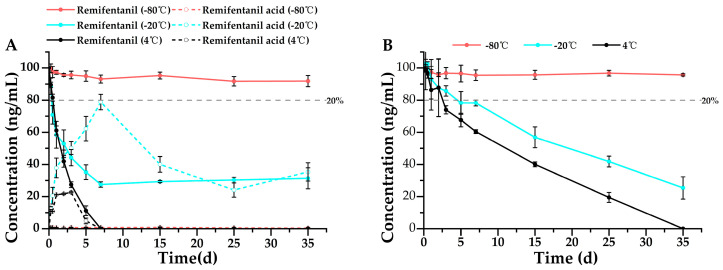
Stability profiles of (**A**) remifentanil (*n* = 3, mean ± SD) and its metabolite (**B**) remifentanil acid (*n* = 3, mean ± SD) in urine stored at 4 °C, −20 °C, and −80 °C. Detailed experimental data on the stability profiles during the first 3 days were presented in Figure S11 of the Supplementary Materials.

**Figure 5:**
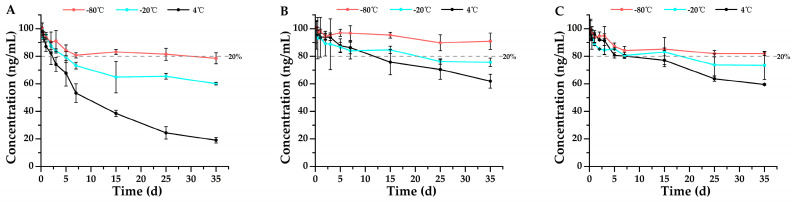
Stability profiles of sufentanil in whole blood stored at three temperatures (4 °C, −20 °C, and −80 °C) using three anticoagulants: (**A**) EDTA-K_2_ (*n* = 3, mean ± SD), (**B**) sodium heparin (*n* = 3, mean ± SD), and (**C**) sodium citrate (*n* = 3, mean ± SD). Detailed experimental data on the stability profiles during the first 3 days were presented in Figure S12 of the Supplementary Materials.

**Figure 6:**
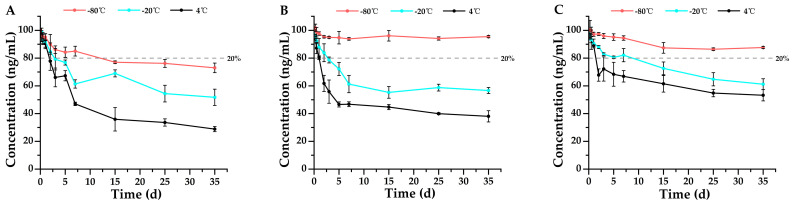
Stability profiles of norsufentanil in whole blood stored at three temperatures (4 °C, −20 °C, and −80 °C) using three anticoagulants: (**A**) EDTA-K_2_ (*n* = 3), (**B**) sodium heparin (*n* = 3), and (**C**) sodium citrate (*n* = 3). Detailed experimental data on the stability profiles during the first 3 days were presented in Figure S13 of the Supplementary Materials.

**Figure 7:**
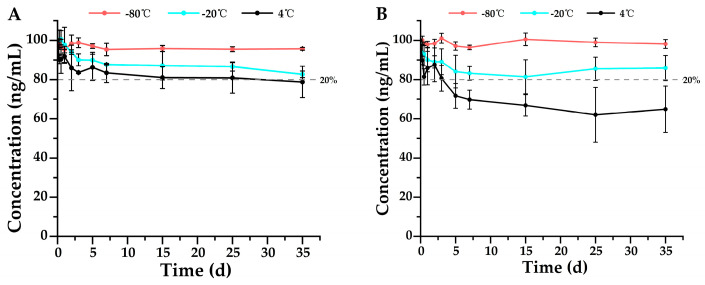
Stability profiles of (**A**) sufentanil (*n* = 3, mean ± SD) and its metabolite (**B**) norsufentanil (*n* = 3, mean ± SD) in urine stored at 4 °C, −20 °C, and −80 °C. Detailed experimental data on the stability profiles during the first 3 days were presented in Figure S14 of the Supplementary Materials.

According to the Forensic Toxicology guidelines for method validation, drugs are considered stable if their concentrations remain within ±20% of the original values, meaning that a degradation of less than 20% is deemed insignificant and falls within the acceptable method bias [20]. This serves as the criterion for stability assessment in the findings of this study. Single-factor ANOVA statistics were performed on all groups of data, and the results were shown in Tables S3–S5 of the Supplementary Materials. The results shown in Figure 2, Figure 3, Figure 4, Figure 5, Figure 6 and Figure 7 are presented as mean ± SD, while the same data were additionally plotted as mean ± 95% confidence intervals (CI) in Figures S15–S19 of the Supplementary Materials.

The stability of remifentanil, sufentanil, and their metabolites in the respective biological matrices was assessed by monitoring changes in their concentrations over storage time in blank spiked whole blood and urine samples under various storage conditions (temperatures and anticoagulants) using the developed HPLC–MS/MS method. As shown in Figure 2A, in whole blood spiked with remifentanil and stored at 4 °C with EDTA-K_2_ as the anticoagulant, remifentanil showed a dramatic decrease in concentration after just 6 h of storage (black line), with a reduction greater than 50%. Furthermore, remifentanil acid (black dotted line) became detectable as early as 6 h, confirming that remifentanil underwent degradation to form its metabolite, remifentanil acid. Moreover, the concentration of remifentanil acid exhibited an initial increase followed by a subsequent decrease over time, verifying the instability of the metabolite as well. When the storage temperature was lowered to −20 °C, the degradation rate of remifentanil decreased slightly, with a loss exceeding 80% occurring after 3 days of storage (blue line); similarly, the degradation rate of remifentanil acid (blue dotted line) also decreased, with its concentration remaining consistently higher from day 5 to day 35 compared to that observed under 4 °C storage conditions. Further reducing the storage temperature to −80 °C led to a significantly slower decrease in remifentanil concentration, with only approximately 20% degradation observed after 15 days (red line); meanwhile, only a minimal amount of remifentanil acid was detected (red dotted line), demonstrating that temperature substantially influences the stability of remifentanil and its metabolites, and that low temperatures can attenuate remifentanil degradation.

Additionally, we evaluated the influence of anticoagulants on the stability of remifentanil in whole blood. In addition to EDTA-K_2_, two other widely used anticoagulants for blood collection were employed: sodium heparin and sodium citrate [21]. Regarding the analysis with sodium heparin (Figure 2B), remifentanil underwent varying degrees of degradation at 4 °C, −20 °C, and −80 °C, all resulting in the production of the metabolite remifentanil acid. However, although the degradation rate of remifentanil remained the slowest at −80 °C, sodium heparin exerted a greater adverse impact on remifentanil stability compared to EDTA-K_2_, with only approximately 60% of the initial concentration retained after 35 days. As depicted in Figure 2C, when sodium citrate was employed as the anticoagulant, the degradation of remifentanil was markedly reduced across all three storage temperatures (4 °C, −20 °C, and −80 °C). Notably, at −80 °C, virtually no degradation occurred after 35 days of storage; at −20 °C, approximately 50% degradation was observed after 35 days; and at 4 °C, about 40% of the initial concentration was retained after 35 days.

Subsequently, the stability of remifentanil’s metabolite, remifentanil acid, in whole blood was investigated, as shown in Figure 3. Unlike the parent drug remifentanil, remifentanil acid demonstrated good stability at −80 °C when EDTA-K_2_ or sodium citrate was used as the anticoagulant (Figure 3A,C). However, remifentanil acid was least stable when sodium heparin served as the anticoagulant, remaining stable for only 7 days even at −80 °C (Figure 3B). Even when stored at 4 °C, remifentanil acid demonstrated a certain degree of stability, remaining stable for 2 days in samples anticoagulated with sodium heparin (Figure 3B), for 5 days in samples anticoagulated with sodium citrate (Figure 3C), and for 7 days in samples anticoagulated with EDTA-K_2_ (Figure 3A). All the above results demonstrated that the metabolite remifentanil acid is more stable in whole blood than its parent drug remifentanil, suggesting that the metabolite has a longer detection window than the parent drug.

Furthermore, the stability of remifentanil and its metabolite, remifentanil acid, in urine was evaluated. Similar to the stability results observed in whole blood, both remifentanil and remifentanil acid remained stable for 35 days at −80 °C in urine (Figure 4). Additionally, both remifentanil and remifentanil acid underwent gradual degradation over time at −20 °C and 4 °C; however, remifentanil acid exhibited superior stability at −20 °C (7 days) and 4 °C (2 days) (Figure 4B) compared to remifentanil (−20 °C: 1 day; 4 °C: 1 day) (Figure 4A). These results further demonstrate that the metabolite is more stable than the parent compound, which is particularly relevant in forensic bioanalysis for drug identification, where greater emphasis should be placed on detecting the metabolite to avoid false negatives resulting from the complete degradation of the parent drug.

#### 3.2.2. Stability of Sufentanil and Its Metabolites Norsufentanil

As shown in Figure 5, sufentanil remained stable for 35 days in whole blood at −80 °C across all three anticoagulants; at −20 °C, it was stable for 5 days in samples anticoagulated with EDTA-K_2_ (Figure 5A), and for 7 days in samples anticoagulated with sodium heparin and sodium citrate (Figure 5B,C); even at 4 °C, sufentanil remained stable for 2 days in samples anticoagulated with EDTA-K_2_, and for 7 days in samples anticoagulated with sodium heparin and sodium citrate. These results indicated that sufentanil is markedly more stable than remifentanil in whole blood and is suitable for storage in tubes anticoagulated with sodium heparin and sodium citrate. Furthermore, although the concentration of sufentanil in whole blood also decreased gradually over time, unlike remifentanil, no metabolite norsufentanil was detected.

As for the stability of sufentanil’s metabolite, norsufentanil, in whole blood (Figure 6), it similarly exhibited the best stability at −80 °C and in samples anticoagulated with sodium citrate (Figure 6C, stable for 7 days at −20 °C). At 4 °C, the stability of norsufentanil was poor across all three anticoagulants, with degradation exceeding 80% after 1 day.

In urine analysis, sufentanil exhibited excellent stability at −20 °C, −80 °C, and even 4 °C, remaining stable for 35 days (Figure 7A), while norsufentanil remained stable at −20 °C and −80 °C, but only for 3 days at 4 °C (Figure 7B). Compared to the stability results in whole blood, sufentanil and norsufentanil showed greater stability in urine samples, suggesting that urine is more suitable as a biological specimen for long-term storage in forensic toxicological analysis and detection of illegal drugs. Similarly, as with sufentanil in whole blood, no metabolite norsufentanil was detected in urine, further demonstrating its chemical stability.

## 4. Discussion

The method addresses the analytical challenges posed by remifentanil acid, which is prone to degradation under alkaline conditions and exhibits poor extraction efficiency with conventional organic solvents. Although Strayer et al. previously reported a solid-phase extraction (SPE)-based LC-MS/MS method for analyzing fentanyl analogs [22], including remifentanil, remifentanil acid, and sufentanil, this approach has practical limitations for high-throughput applications due to high costs, procedural complexity, and limited sample throughput associated with column-based SPE methodologies. The use of protein precipitation, along with fentanyl-d_5_ as a single isotope-labeled internal standard (IS), is a well-established and reliable laboratory practice. Our findings confirm that fentanyl-d_5_ effectively normalized the recoveries and matrix effects of all four analytes, demonstrating its suitability for this application. This approach provides a more efficient and consistent solution for accurate quantification, overcoming the limitations associated with conventional methods.

The recoveries above 110% observed in urine were attributable to mild ion enhancement, which is common in electrospray LC–MS/MS due to the diverse endogenous components in urine [23,24]. Additionally, using pooled matrix-matched calibration curves along with the isotopic IS fentanyl-d_5_ effectively corrected these matrix-related shifts. After normalization, both accuracy (87.83–115.21%) and precision (RSD ≤ 13.57%) fell within the acceptance criteria of the FDA guideline [25]. All the validation results confirm that the developed HPLC–MS/MS method is robust, demonstrating adequate sensitivity and meeting the requirements for bioanalytical applications.

The evaluation of the stability of drugs, as well as that of their metabolites, is a critically important parameter in the determination of analytes in biological matrices in drug metabolism, pharmacokinetics, toxicological, clinical, and forensic studies [26]. Especially in forensic toxicology, the determined concentrations of the detected analytes should accurately reflect those at the time of collection or, for postmortem samples, at the time of death [27]. However, in general, quantification of the analytes is not performed immediately after sample collection, during which analyte degradation might occur during storage prior to analysis. Consequently, incorrect quantification resulting from the instability of drugs and their metabolites can have a significant impact on medico-legal investigations. Thus, it is essential to evaluate the stability of analytes and their metabolites under various storage conditions, particularly because metabolites persist longer in vivo than their parent drugs—a feature that is especially valuable for forensic toxicology applications, such as identifying illicit drug use in forensic cases [28]. As fentanyl analogs, remifentanil and sufentanil have been reported to exhibit instability in blood and urine matrices, while the stability of their metabolites remains poorly characterized.

Although light exposure, controlled pH titration, and container material may also influence stability, these factors are largely mitigated in clinical and forensic contexts by standardized laboratory storage guidelines and certified commercial collection tubes. The present study focused on the three pre-analytical factors most frequently responsible for opioid instability in routine practice—temperature, matrix, and anticoagulant—because these variables introduce unpredictable degradation during real specimen handling. EDTA-K_2_, sodium heparin, and sodium citrate were selected because they are the most widely used anticoagulants in clinical diagnostics and forensic toxicology [29,30,31,32]. Thus, different storage temperatures (refrigerated at 4 °C, frozen at −20 °C, and ultra-low at −80 °C) and various anticoagulants (sodium heparin, EDTA-K_2_, and sodium citrate) were selected to systematically evaluate the stability of remifentanil, sufentanil, and their metabolites (remifentanil acid and norsufentanil) in blood and urine samples. 

Based on the stability test results for remifentanil in whole blood at 4 °C, −20 °C, and −80 °C with the anticoagulants EDTA-K_2_, sodium heparin, and sodium citrate (Figure 2), remifentanil exhibited the highest stability at −80 °C in the presence of sodium citrate, followed by moderate stability in the presence of EDTA-K_2_; in contrast, it was unstable across all three storage temperatures in the presence of sodium heparin. Furthermore, under all three anticoagulants, the stability at 4 °C and −20 °C was limited to within 1 day. The results were consistent with previous studies, which demonstrated that remifentanil is highly unstable during storage at 4 °C in whole blood samples, with a significant decrease in concentration (>50%) observed after one day [16]. Remifentanil degrades rapidly in whole blood because its ester linkage is readily hydrolyzed by circulating nonspecific esterases, primarily carboxylesterases and cholinesterases, with activity strongly influenced by the local pH [33]. Accordingly, the choice of anticoagulant shapes the microenvironment in which hydrolysis occurs. Sodium citrate generates a defined citrate–citric acid buffer that maintains a mildly acidic and chemically stable milieu, thereby attenuating esterase activity and limiting further pH drift during storage [34]. In contrast, EDTA provides divalent-ion chelation with minimal sustained buffering, and heparin preserves a near-neutral environment; both conditions support continued esterase activity and thus faster opioid hydrolysis [35,36]. The concurrent degradation of remifentanil and formation of remifentanil acid observed across matrices further supports esterase-mediated hydrolysis as the dominant degradation pathway. Moreover, Koster et al. found that remifentanil was particularly unstable in untreated EDTA-anticoagulated whole blood, while remifentanil’s stability can be increased in acidified EDTA plasma, suggesting that acidic conditions may inhibit enzymatic degradation, thus enhancing stability [9]. Jung et al. also reported instability of remifentanil in refrigerated whole blood and recommended frozen storage for reliable quantification. Other recent studies emphasize that avoiding alkaline exposure, maintaining low temperatures, and ensuring prompt processing are critical to preventing artifactual loss of remifentanil. This finding aligns with our results (Table S6), where sodium citrate’s mildly acidic pH may slightly limit esterase activity, leading to the highest stability of remifentanil [8]. On the other hand, remifentanil is extremely unstable in strong and moderately basic conditions [16]. Sodium heparin, with a pH around 7, may preserve esterase integrity, resulting in the poorest stability of remifentanil under the storage conditions tested. Thus, the differences in remifentanil stability across different anticoagulants may be attributed to their influence on pH and esterase activity, which are key drivers of remifentanil hydrolysis.

Sufentanil is 500–800 times more potent than morphine, and its side effects and addiction potential make its quantification particularly significant in clinical drug monitoring and forensic toxicological analysis. It is therefore critical to ensure the stability of sufentanil in biological samples before analysis; however, there is little literature reporting on the stability of sufentanil in biological samples, and studies on its metabolites are almost nonexistent. Dufresne et al. reported rapid loss of sufentanil at 4 °C in nonsilanized tubes (degradation within hours), consistent with our observation that room/refrigerated conditions are suboptimal for long-term preservation unless special measures (e.g., immediate freezing or appropriate containers) are used. More recent long-term work has raised caution that even frozen storage can show concentration decreases over many years [11]; Wehrfritz and colleagues reported substantial decreases in sufentanil concentrations after extended storage at −20 °C over multi-year periods [12]. In this study, the stability profiles of sufentanil and its metabolite norsufentanil in whole blood and urine samples were tested under the same conditions as those for remifentanil and remifentanil acid. This further demonstrates the chemical instability of remifentanil, which contains an ester bond and undergoes ester hydrolysis in vitro to form remifentanil acid, whereas sufentanil generally has greater stability than remifentanil and good preservation at −80 °C—also consistent with earlier observations. In this study, sufentanil remained comparatively stable across matrices and anticoagulants at −80 °C (stable through 35 days in whole blood and urine in our experiments), whereas at higher temperatures (−20 °C and 4 °C), gradual loss was observed depending on the anticoagulant. Notably, unlike remifentanil, we did not detect a stable accumulation of a major sufentanil metabolite (norsufentanil) in the same manner as remifentanil acid appeared for remifentanil. This is consistent with the literature, which indicates different metabolic and chemical stability behaviors between ester-containing (remifentanil) and non-ester opioids (sufentanil).

Following the investigation of the stability of remifentanil, sufentanil, and their respective metabolites, remifantanil acid and norsufentanil, under near-realistic storage conditions (refrigerated at 4 °C, frozen at −20 °C, and ultra-low at −80 °C) with anticoagulation using EDTA-K_2_, sodium heparin, or sodium citrate, it is recommended that, where feasible, remifentanil, sufentanil, remifantanil acid, and norsufentanil be stored at −80 °C, with sodium citrate-anticoagulated tubes being more suitable for preservation. Remifentanil should not be stored in whole blood or urine at −20 °C for more than 1 day; consequently, remifentanil acid may serve as a reliable biomarker when immediate cryopreservation of remifentanil is not feasible. Sufentanil showed pronounced resistance to degradation relative to remifentanil, which in whole blood at −20 °C for 5 days and in urine at −20 °C for up to 35 days. Overall, neither whole blood nor urine samples containing remifentanil or sufentanil should be stored at 4 °C for extended periods, and samples should be analyzed as soon as possible after collection. In addition, several practical considerations should be noted. When immediate −80 °C freezing is not feasible, urine represents a more stable matrix for remifentanil and can therefore be used preferentially, whereas citrate-anticoagulated blood is advisable if blood collection is unavoidable. For sufentanil, both blood and urine are acceptable matrices, provided that samples are promptly cooled, frozen without delay. Regardless of matrix, long-term storage at −80 °C remains recommended. In operational settings such as ambulance-based sampling, emergency departments, or field investigations, prioritizing urine collection when possible, and transferring them to −80 °C at the earliest opportunity can further help maintain analytical reliability.

In this study, there exist several limitations. Several aspects warrant further investigation in this study. Freeze–thaw behavior was not examined, and the targeted MRM approach focused only on two known metabolites, remifentanil acid and norsufentanil. These elements will be explored in greater depth in future work.

## 5. Conclusions

A sensitive and fully validated HPLC-MS/MS method was established for the simultaneous quantification of remifentanil, sufentanil, and their major metabolites in human whole blood and urine. Systematic stability evaluation revealed substantial differences in analyte preservation across storage conditions. Remifentanil degraded rapidly in blood, particularly at 4 °C, underscoring the necessity of immediate storage at –80 °C and the use of sodium citrate as an anticoagulant to inhibit esterase-mediated hydrolysis. In contrast, remifentanil acid demonstrated considerably greater stability, supporting its utility as a primary biomarker for an extended detection window. Sufentanil and norsufentanil exhibited stronger overall stability, with optimal preservation achieved at –80 °C; however, norsufentanil degraded quickly at 4 °C. Based on these results, it is recommended that biological samples containing these fentanyl analogues be stored at –80 °C in sodium citrate-treated containers to maintain analyte integrity. These results underscore the importance of immediate low-temperature storage and appropriate anticoagulant selection in enhancing the accuracy of forensic and clinical analyses, by providing practical recommendations to mitigate degradation risks, particularly when sample processing is delayed.

## Figures and Tables

**Table 1:** MRM parameters for remifentanil, sufentanil, and their metabolites.

Compound	Precursor Ion	Product Ion	Fragmentor/v	CE/v
Remifentanil	377.1	113.1 *	135	30
		317.1		15
Remifentanil acid	363.1	113.1 *	130	30
		259.1		15
Sufentanil	387.1	238.1 *	130	20
		355.1		18
Norsufentanil	277.1	96.2 *	105	23
		128.2		11
Fentanyl-d_5_(IS)	342.2	188.2 *	125	25
		105.2		45

* Quantitative ion.

## Data Availability

Data are available from the authors upon reasonable request.

## Supplementary Materials

The following supporting information can be downloaded at: https://www.mdpi.com/article/10.3390/metabo15120804/s1, Figure S1. HPLC-MS/MS MRM chromatogram and mass spectra for remifentanil in a spiked blood sample. Figure S2. HPLC-MS/MS MRM chromatogram and mass spectra for remifentanil acid in a spiked blood sample. Figure S3. HPLC-MS/MS MRM chromatogram and mass spectra for sufentanil in a spiked blood sample. Figure S4. HPLC-MS/MS MRM chromatogram and mass spectra for norsufentanil in a spiked blood sample. Figure S5. HPLC-MS/MS MRM chromatogram and mass spectra for remifentanil in a spiked urine sample. Figure S6. HPLC-MS/MS MRM chromatogram and mass spectra for remifentanil acid in a spiked urine sample. Figure S7. HPLC-MS/MS MRM chromatogram and mass spectra for sufentanil in a spiked urine sample. Figure S8. HPLC-MS/MS MRM chromatogram and mass spectra for norsufentanil in a spiked urine sample. Figure S9. Stability profiles of remifentanil in whole blood stored at three temperatures (4 °C, −20 °C, and −80 °C) using three anticoagulants: (A) EDTA-K_2_ (*n* = 3, mean ± SD), (B) sodium heparin (*n* = 3, mean ± SD), and (C) sodium citrate (*n* = 3, mean ± SD) over the first 3 days. Figure S10. Stability profiles of remifentanil acid in whole blood stored at three temperatures (4 °C, −20 °C, and −80 °C) using three anticoagulants: (A) EDTA-K_2_ (*n* = 3, mean ± SD), (B) sodium heparin (*n* = 3, mean ± SD), and (C) sodium citrate (*n* = 3, mean ± SD) over the first 3 days. Figure S11. Stability profiles of (A) remifentanil (*n* = 3, mean ± SD) and its metabolite (B) remifentanil acid (*n* = 3, mean ± SD) in urine stored at 4 °C, −20 °C, and −80 °C over the first 3 days. Figure S12. Stability profiles of sufentanil in whole blood stored at three temperatures (4 °C, −20 °C, and −80 °C) using three anticoagulants: (A) EDTA-K_2_ (*n* = 3, mean ± SD), (B) sodium heparin (*n* = 3, mean ± SD), and (C) sodium citrate (*n* = 3, mean ± SD) over the first 3 days. Figure S13. Stability profiles of norsufentanil in whole blood stored at three temperatures (4 °C, −20 °C, and −80 °C) using three anticoagulants: (A) EDTA-K_2_ (*n* = 3, mean ± SD), (B) sodium heparin (*n* = 3, mean ± SD), and (C) sodium citrate (*n* = 3, mean ± SD) over the first 3 days. Figure S14. Stability profiles of (A) sufentanil (*n* = 3, mean ± SD) and its metabolite (B) norsufentanil (*n* = 3, mean ± SD) in urine stored at 4 °C, −20 °C, and −80 °C over the first 3 days. Figure S15. Stability profiles of remifentanil in whole blood stored at three temperatures (4 °C, −20 °C, and −80 °C) using three anticoagulants: (A) EDTA-K_2_ (*n* = 3, mean ± 95% CI), (B) sodium heparin (*n* = 3, mean ± 95% CI), and (C) sodium citrate (*n* = 3, mean ± 95% CI). Figure S16. Stability profiles of remifentanil acid in whole blood stored at three temperatures (4 °C, −20 °C, and −80 °C) using three anticoagulants: (A) EDTA-K_2_ (*n* = 3, mean ± 95% CI), (B) sodium heparin (*n* = 3, mean ± 95% CI), and (C) sodium citrate (*n* = 3, mean ± 95% CI). Figure S17. Stability profiles of sufentanil in whole blood stored at three temperatures (4 °C, −20 °C, and −80 °C) using three anticoagulants: (A) EDTA-K_2_ (*n* = 3, mean ± 95% CI), (B) sodium heparin (*n* = 3, mean ± 95% CI), and (C) sodium citrate (*n* = 3, mean ± 95% CI). Figure S18. Stability profiles of norsufentanil in whole blood stored at three temperatures (4 °C, −20 °C, and −80 °C) using three anticoagulants: (A) EDTA-K_2_ (*n* = 3, mean ± 95% CI), (B) sodium heparin (n = 3, mean ± 95% CI), and (C) sodium citrate (*n* = 3, mean ± 95% CI). Figure S19. Stability profiles of (A) sufentanil (*n* = 3, mean ± 95% CI) and its metabolite (B) norsufentanil (*n* = 3, mean ± 95% CI) in urine stored at 4 °C, −20 °C, and −80 °C. Table S1. Standard calibration curves of remifentanil, sufentanil, and their metabolites (remifentanil acid and norsufentanil) in whole blood and urine samples. Table S2. Accuracy, precision, matrix effect, and extraction recovery rate of remifentanil, sufentanil, and their metabolites in whole blood and urine samples (*n* = 6). Table S3. One-way ANOVA with pairwise comparisons of blood samples stored at different temperatures within the same anticoagulant. Table S4. One-way ANOVA with pairwise comparisons of blood samples containing different anticoagulants under the same temperature condition. Table S5. One-way ANOVA with pairwise comparisons of urine samples stored at three different temperatures. Table S6. Measurement results of pH value of the samples (*n* = 6).

## Abbreviations

The following abbreviations are used in this manuscript:

HPLC-MS/MS	High-performance liquid chromatography–tandem mass spectrometry
RSD	Relative standard deviation
LC-MS/MS	Liquid chromatography–tandem mass spectrometry
EDTA-K_2_	Edathamil
MRM	Multiple reaction monitoring
IS	Internal standard
UV	Ultraviolet
GC-MS	Gas chromatography–tandem mass spectrometry
LOD	Limits of detection
Rt	Retention time
SD	Standard deviation
CI	confidence intervals
