# Supplementary material for: Study on Stability of Remifentanil, Sufentanil, and Their Metabolites in Human Whole Blood and Urine
Source: Metabolites. 2025 Dec 18;15(12):804. doi: 10.3390/metabo15120804 (PMC12734456; doi:10.3390/metabo15120804)
Supplement: Supplementary file 1 [file metabolites-15-00804-s001.zip › metabolites-4013610-supplementary.pdf]

SUPPLEMENTARY MATERIAL

Study on Stability of Remifentanil, Sufentanil, and Their Metabolites in Human Whole Blood and Urine

Zhuoyi Wang[1,2,3], Huan Gao[1,2,3], Yingwen Xu[1,2,3], Di Liang[1,2,3], Xian Ju[1,2,3], Kaili Du[1,2,3,5], Xiaoxi Mu[1,6], Xi Zhang[1,2,3], Ziyang Dong[1,2,3], Tao Wang[1,2,3], Dan Zhang[1,2,3], Zhiwen Wei[1,2,3], Jianguo Li[4], Keming Yun[1,2,3,*], and Zhe Chen[1,2,3,4*]

[1] School of Forensic Medicine, Shanxi Medical University, Jinzhong 030600, China;
[2] Shanxi Key Laboratory of Forensic Medicine, Jinzhong 030600, China;
[3] Key Laboratory of Forensic Toxicology of Ministry of Public, Jinzhong 030600, China;
[4] China Institute for Radiation Protection, Taiyuan 030001, China
[5] Department of Pathology, Shanxi Medical University, Taiyuan 030001, China;
[6] Taiyuan Railway Public Security Bureau Linfen Public Security Department, Linfen 041000, China;
* Correspondence: yunkeming5142@163.com (K.Y.); chenzhe0322@163.com (Z.C.).

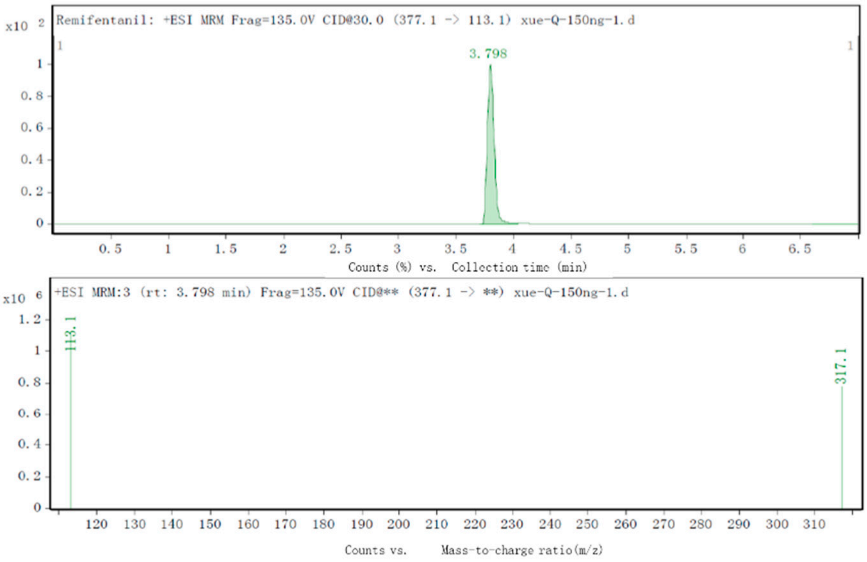

Figure S1. HPLC-MS/MS MRM chromatogram and mass spectra for remifentanil in a spiked blood sample.

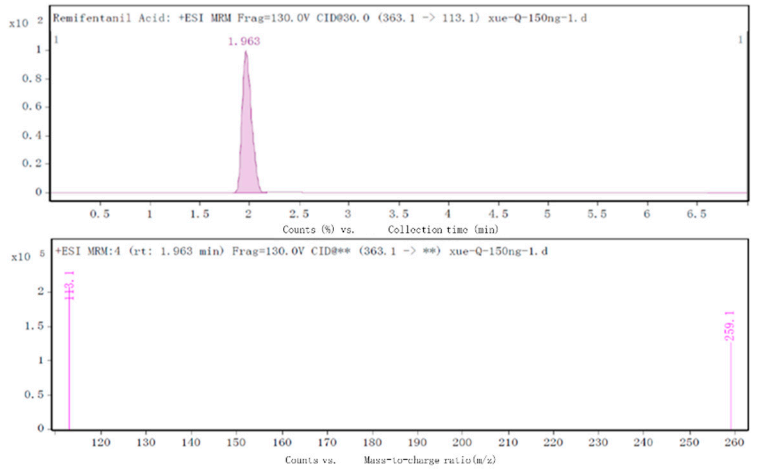

Figure S2. HPLC-MS/MS MRM chromatogram and mass spectra for remifentanil acid in a spiked blood sample.

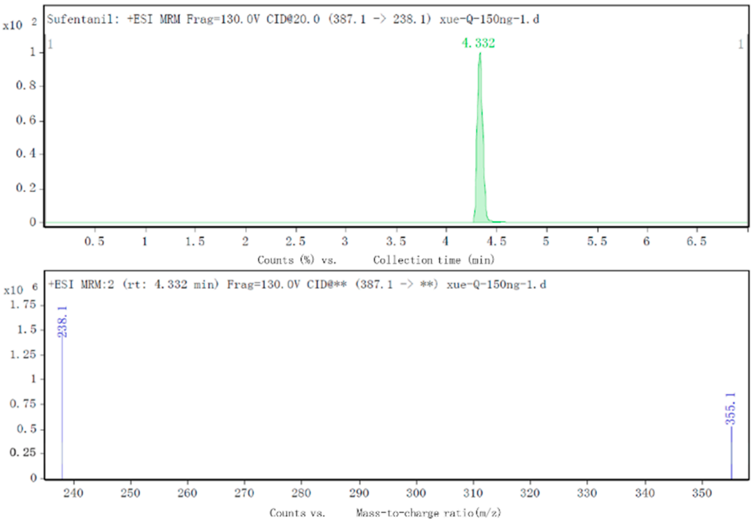

Figure S3. HPLC-MS/MS MRM chromatogram and mass spectra for sufentanil in a spiked blood sample.

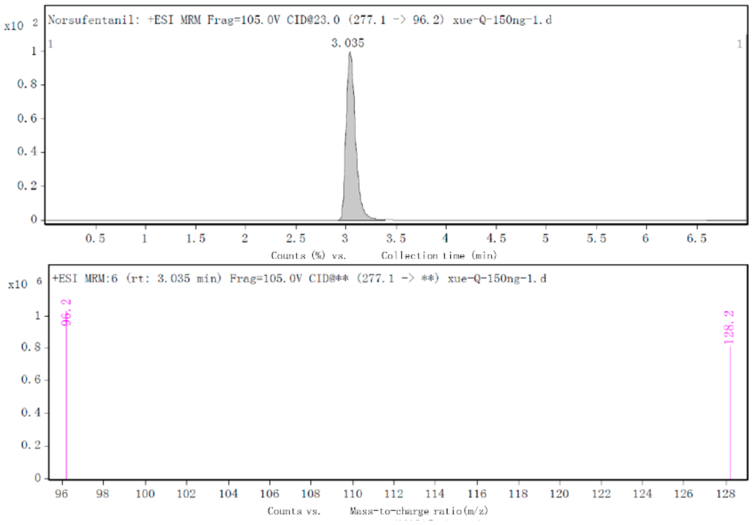

Figure S4. HPLC-MS/MS MRM chromatogram and mass spectra for norsufentanil in a spiked blood sample.

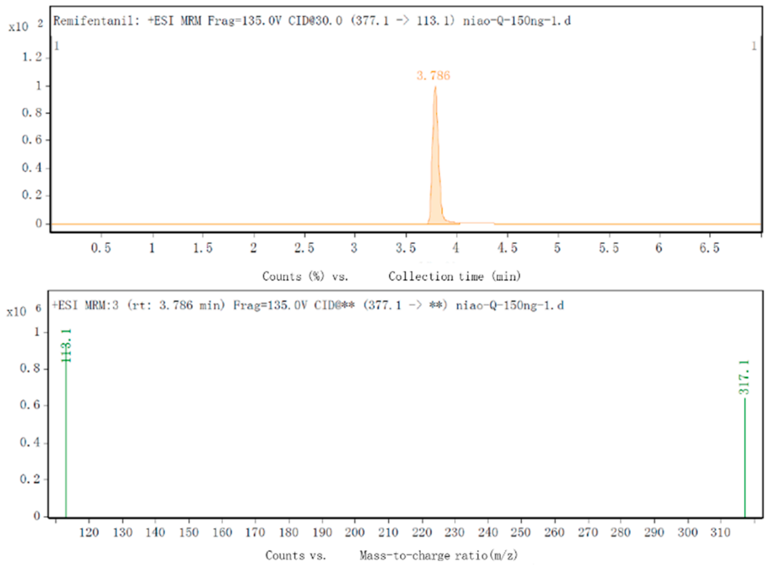

Figure S5. HPLC-MS/MS MRM chromatogram and mass spectra for remifentanil in a spiked urine sample.

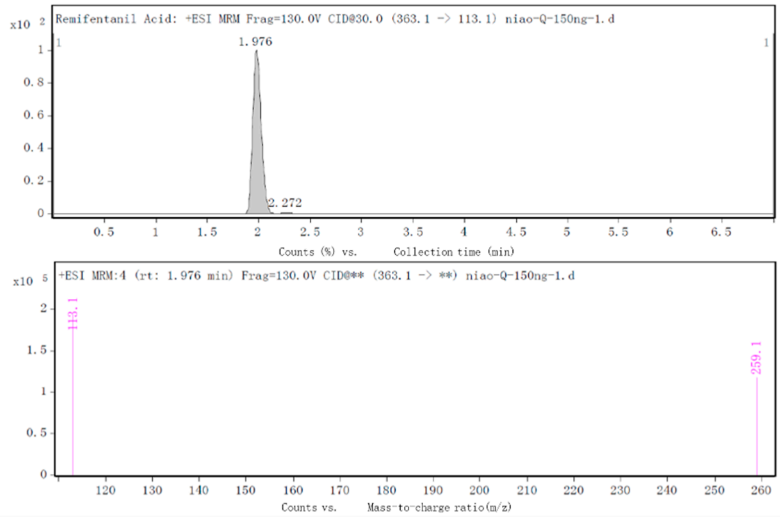

Figure S6. HPLC-MS/MS MRM chromatogram and mass spectra for remifentanil acid in a spiked urine sample.

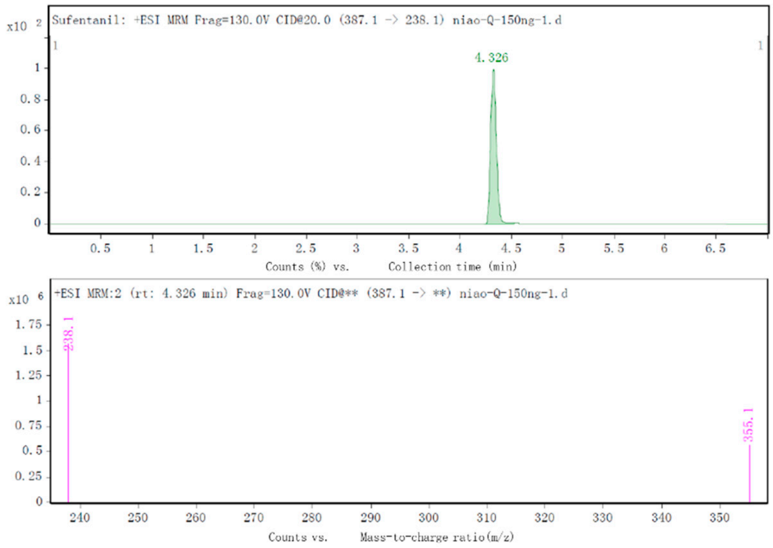

Figure S7. HPLC-MS/MS MRM chromatogram and mass spectra for sufentanil in a spiked urine sample.

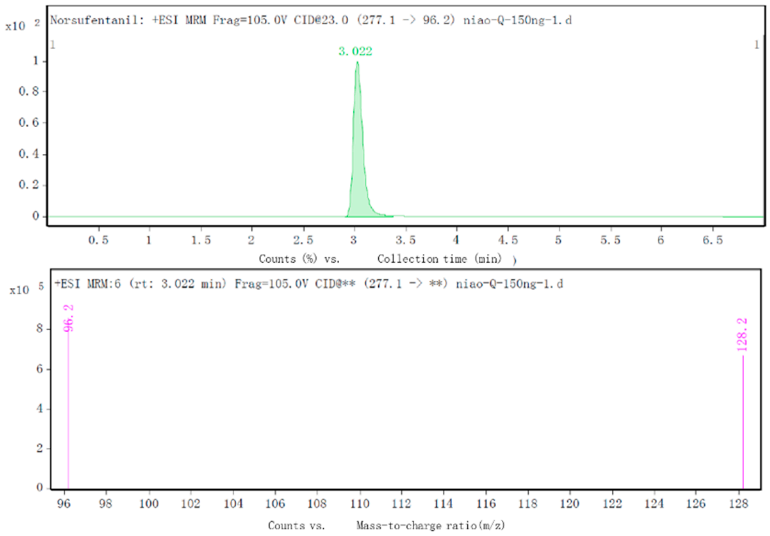

Figure S8. HPLC-MS/MS MRM chromatogram and mass spectra for norsufentanil in a spiked urine sample.

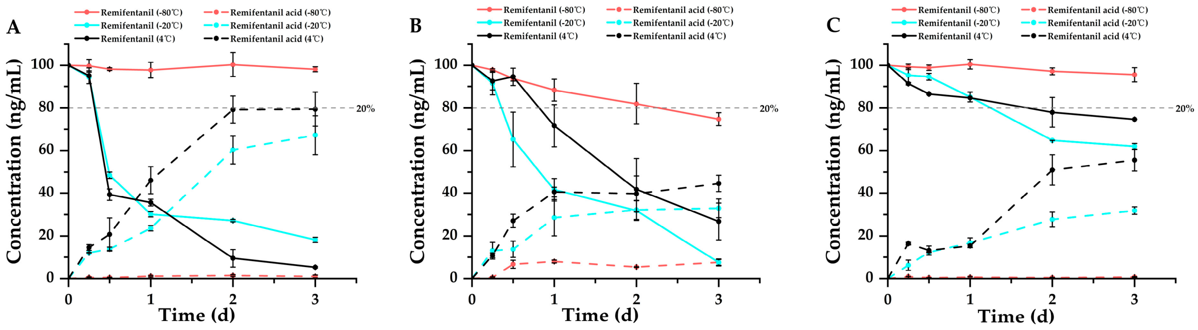

Figure S9. Stability profiles of remifentanil in whole blood stored at three temperatures (4 °C, −20 °C, and −80 °C) using three anticoagulants: (A) EDTA-K_2 ($n = 3$, mean ± SD), (B) sodium heparin ($n = 3$, mean ± SD), and (C) sodium citrate ($n = 3$, mean ± SD) over the first 3 days.

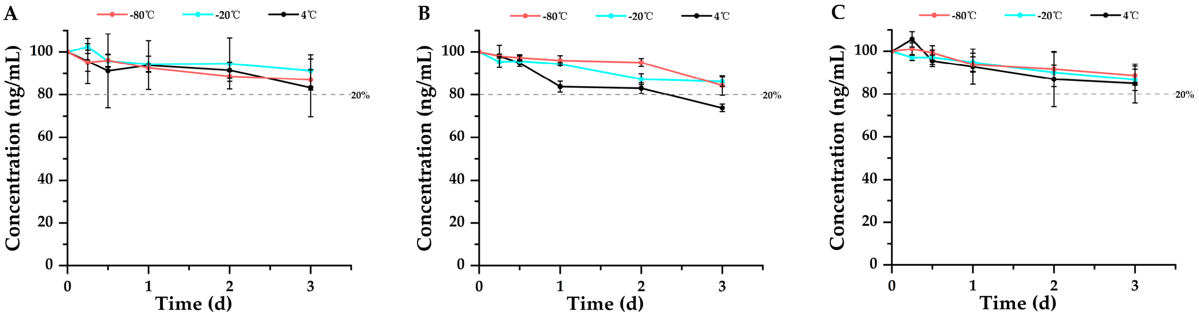

Figure S10. Stability profiles of remifentanil acid in whole blood stored at three temperatures (4 °C, −20 °C, and −80 °C) using three anticoagulants: (A) EDTA-K_2 (n = 3, mean ± SD), (B) sodium heparin (n = 3, mean ± SD), and (C) sodium citrate (n = 3, mean ± SD) over the first 3 days.

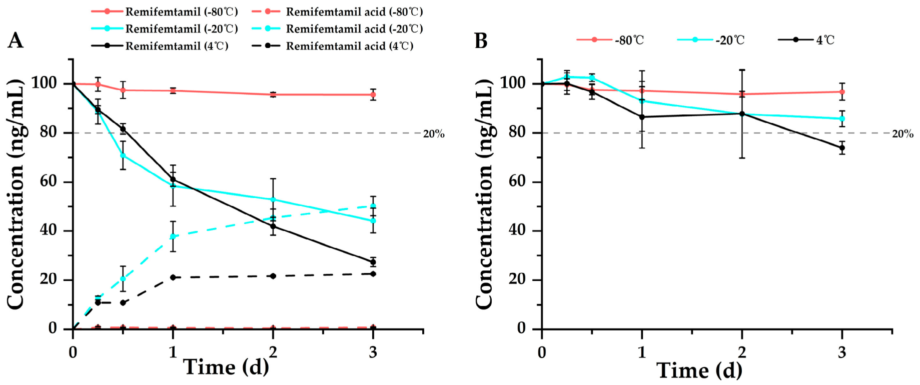

Figure S11. Stability profiles of (A) remifentanil (n = 3, mean ± SD) and its metabolite (B) remifentanil acid (n = 3, mean ± SD) in urine stored at 4 °C, −20 °C, and −80 °C over the first 3 days.

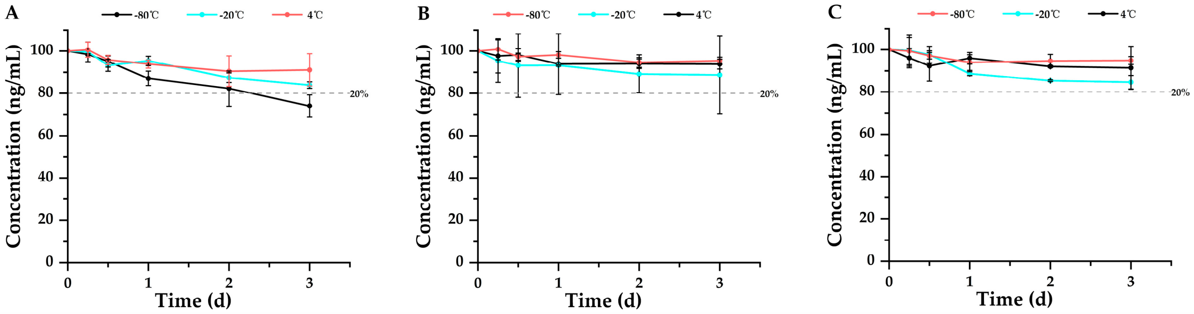

Figure S12. Stability profiles of sufentanil in whole blood stored at three temperatures (4 °C, −20 °C, and −80 °C) using three anticoagulants: (A) EDTA-K_2 (n = 3, mean ± SD), (B) sodium heparin (n = 3, mean ± SD), and (C) sodium citrate (n = 3, mean ± SD) over the first 3 days.

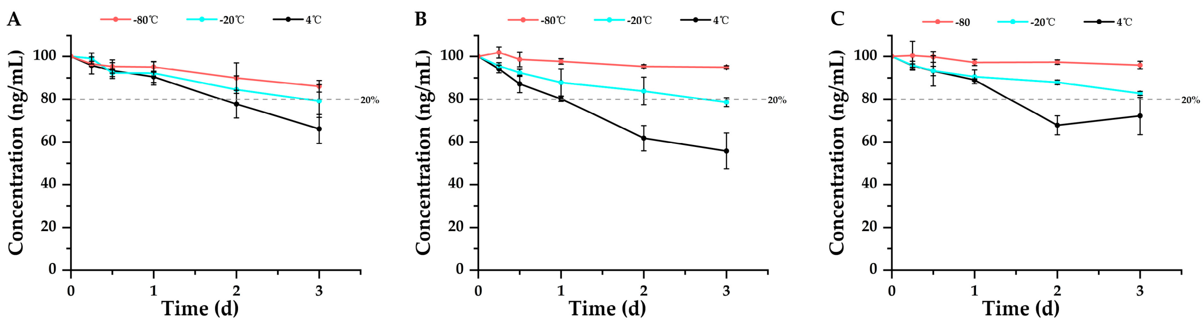

Figure S13. Stability profiles of norsufentanil in whole blood stored at three temperatures (4 °C, −20 °C, and −80 °C) using three anticoagulants: (A) EDTA-K_2 (n = 3, mean ± SD), (B) sodium heparin (n = 3, mean ± SD), and (C) sodium citrate (n = 3, mean ± SD) over the first 3 days.

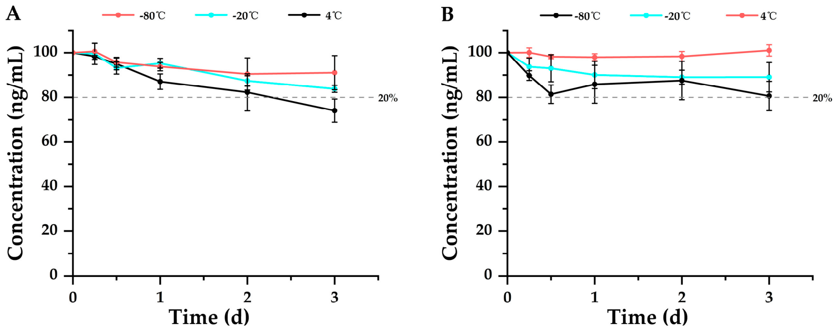

Figure S14. Stability profiles of (A) sufentanil (n = 3, mean ± SD) and its metabolite (B) norsufentanil (n = 3, mean ± SD) in urine stored at 4 °C, −20 °C, and −80 °C over the first 3 days.

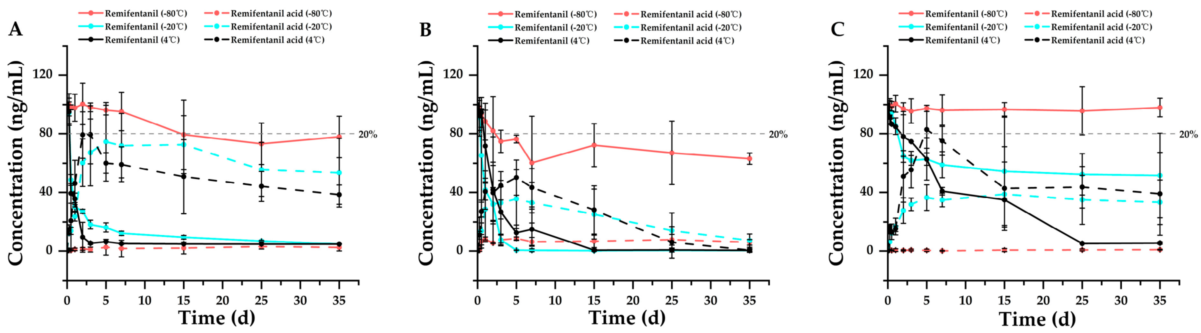

Figure S15. Stability profiles of remifentanil in whole blood stored at three temperatures (4 °C, −20 °C, and −80 °C) using three anticoagulants: (A) EDTA-K_2 (n = 3, mean ± 95% CI), (B) sodium heparin (n = 3, mean ± 95% CI), and (C) sodium citrate (n = 3, mean ± 95% CI).

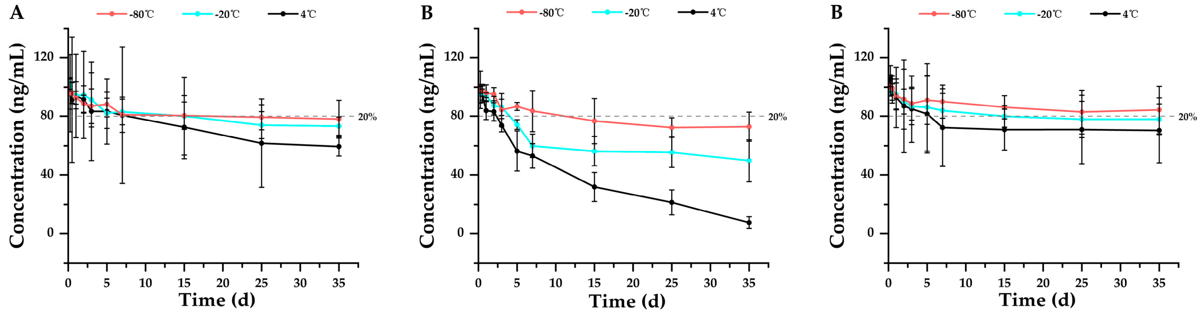

Figure S16. Stability profiles of remifentanil acid in whole blood stored at three temperatures (4 °C, −20 °C, and −80 °C) using three anticoagulants: (A) EDTA-K_2 (n = 3, mean ± 95% CI), (B) sodium heparin (n = 3, mean ± 95% CI), and (C) sodium citrate (n = 3, mean ± 95% CI).

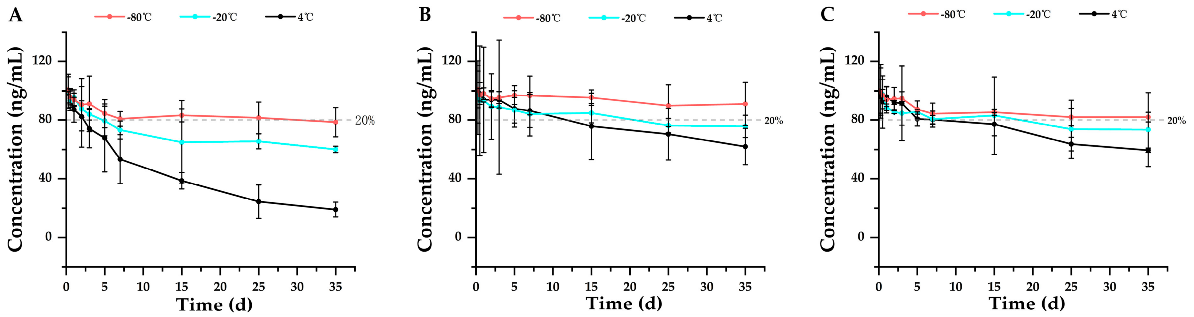

Figure S17. Stability profiles of sufentanil in whole blood stored at three temperatures (4 °C, −20 °C, and −80 °C) using three anticoagulants: (A) EDTA-K_2 (n = 3, mean ± 95% CI), (B) sodium heparin (n = 3, mean ± 95% CI), and (C) sodium citrate (n = 3, mean ± 95% CI).

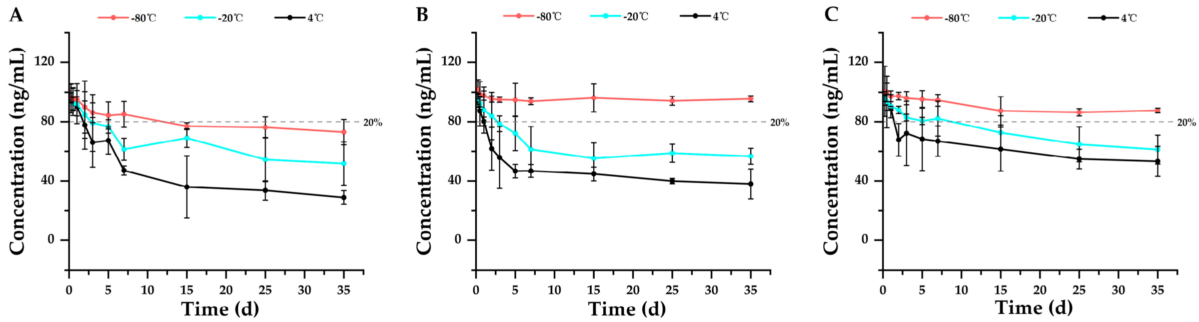

Figure S18. Stability profiles of norsufentanil in whole blood stored at three temperatures (4 °C, −20 °C, and −80 °C) using three anticoagulants: (A) EDTA-K_2 (n = 3, mean ± 95% CI), (B) sodium heparin (n = 3, mean ± 95% CI), and (C) sodium citrate (n = 3, mean ± 95% CI).

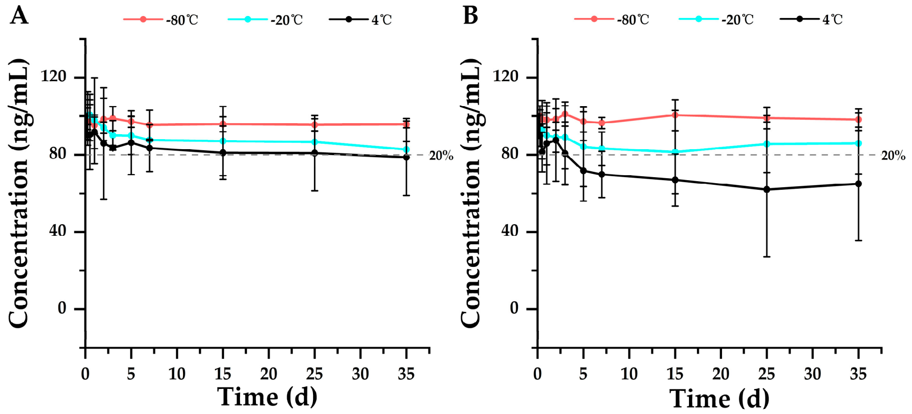

Figure S19. Stability profiles of (A) sufentanil (n = 3, mean ± 95% CI) and its metabolite (B) norsufentanil (n = 3, mean ± 95% CI) in urine stored at 4 °C, −20 °C, and −80 °C.

Table S1. Standard calibration curves of remifentanil, sufentanil and their metabolites (remifentanil acid and norsufentanil) in whole blood and urine samples.

Compound	Matrix	Linear equation	Linearity range (ng/mL)	Correlation coefficient (r^2)	LOD (ng/mL)
Remifentanil	blood	y=0.691x + 0.003	0.10~200.00	0.999	0.06
	urine	y=0.609x + 0.053	0.10~200.00	0.998	0.02
Remifentanil acid	blood	y=0.022x + 0.002	0.10~200.00	0.999	0.20
	urine	y=0.013x +0.009	0.10~200.00	0.998	0.04
Sufentanil	blood	y=0.891x + 0.009	0.10~200.00	0.999	0.03
	urine	y=0.968x + 0.071	0.10~200.00	0.999	0.01
Norsufentanil	blood	y=0.972x + 0.007	0.10~200.00	0.999	0.02
	urine	y=0.360x + 0.035	0.10~200.00	0.998	0.02

Table S2. Accuracy, precision, matrix effect and extraction recovery rate of remifentanil, sufentanil and their metabolites in whole blood and urine samples (n = 6).

Compound	Matrix	Concentration (ng/mL)	Matrix effect (%)	Extraction recovery rate (%)	Accuracy (RSD%)		Precision (RSD%)	
					Inter-assay	Intra-assay	Inter-assay	Intra-assay
Remifentanil	blood	0.50	88.41	102.56	94.40	89.35	10.98	10.58
		20.00	94.15	100.83	93.12	106.67	12.72	7.05
		150.00	92.92	111.14	96.06	95.96	0.23	4.37
	urine	0.50	88.49	95.50	95.43	89.27	4.78	8.76
		20.00	95.66	99.58	110.89	100.99	5.14	9.02
		150.00	86.71	96.36	106.35	109.20	7.85	3.63
Remifentanil acid	blood	0.50	103.75	118.72	105.72	107.16	2.95	13.56
		20.00	104.13	101.62	99.52	96.92	7.07	4.24
		150.00	92.67	105.28	105.71	94.41	2.73	12.27
	urine	0.50	94.51	99.55	106.30	100.57	3.22	10.65
		20.00	85.06	99.62	107.92	113.68	3.98	5.39
		150.00	87.73	93.90	112.76	109.18	7.50	13.05
Sufentanil	blood	0.50	90.68	101.54	94.32	89.87	9.71	12.34
		20.00	101.16	100.68	97.67	106.59	9.59	3.42
		150.00	95.06	109.13	98.28	96.90	6.22	4.71
	urine	0.50	106.30	119.42	99.89	89.84	5.81	11.44
		20.00	113.44	100.146	106.99	97.47	6.78	13.57
		150.00	112.20	95.46	110.34	108.27	3.13	8.00
Norsufentanil	blood	0.50	101.02	100.54	97.10	87.83	0.29	8.62
		20.00	106.93	95.20	97.58	105.92	0.68	1.20
		150.00	98.76	102.55	95.63	102.00	0.42	4.37
	urine	0.50	93.65	107.47	102.13	97.33	1.54	5.56
		20.00	86.34	100.10	104.04	115.21	3.01	6.54
		150.00	88.21	92.63	110.83	106.79	2.68	7.97

*: $p < 0.05$; **: $p < 0.01$

Table S3. One-way ANOVA with pairwise comparisons of blood samples stored at different temperatures within the same anticoagulant.

Compounds	Anticoagulant	Sum of squares	Mean square	F	*p*-value
	EDTA	27919.73	13959.87	16.17	0.00**
Remifentanil	Sodium heparin	14428.24	7214.12	6.49	0.00**
	Sodium citrate	8146.95	4073.48	8.16	0.00**
Remifentanil	EDTA	173.927	86.96	0.75	0.48
acid	Sodium heparin	3080.83	1540.41	3.04	0.06
	Sodium citrate	2268.28	1134.14	5.12	0.02*
	EDTA	241.53	120.77	1.15	0.33
Sufentanil	Sodium heparin	525.52	262.76	3.49	0.04*
	Sodium citrate	2728.61	1364.30	3.60	0.04*
	EDTA	2257.17	1128.59	3.07	0.06
Norsufentanil	Sodium heparin	6217.76	3108.88	11.47	0.00**
	Sodium citrate	2205.87	1102.93	7.11	0.00**

*: $p< 0.05$; **: $p< 0.01$

Table S4. One-way ANOVA with pairwise comparisons of blood samples containing different anticoagulants under the same temperature condition.

Compounds	Temperature	Sum of squares	Mean square	F	*p*-value
	-80℃	1911.914	955.96	9.59	0.00**
Remifentanil	-20℃	11134.68	5567.34	5.58	0.00**
	4℃	5748.936	2874.47	2.09	0.14
Remifentanil	-80℃	118.626	59.313	0.87	0.43
acid	-20℃	1821.92	910.96	9.31	0.02*
	4℃	2937.16	1468.58	3.16	0.05*
	-80℃	282.41	141.21	3.48	0.04*
Sufentanil	-20℃	2569.83	1284.91	3.20	0.05*
	4℃	207.381	103.69	0.88	0.42
	-80℃	553.38	276.69	7.10	0.00**
Norsufentanil	-20℃	234.24	117.12	0.48	0.62
	4℃	769.41	384.71	0.75	0.48

*: $p< 0.05$; **: $p< 0.01$

Table S5. One-way ANOVA with pairwise comparisons of urine samples stored at three different temperatures.

Compounds	Sum of squares	Mean square	F	*p*-value
Remifentanil	20262.36	10131.18	13.79	0.00**
Remifentanil acid	5280.59	2640.29	4.409	0.02*
Sufentanil	602.61	301.30	11.06	0.00**
Norsufentanil	2328.70	1164.35	19.73	0.00**

*: $p< 0.05$; **: $p< 0.01$

Table S6. Measurement results of pH value of the samples（n = 6)

Sample	1	2	3	4	5	6	Mean
Urine	6.71	6.52	5.78	5.91	5.51.	6.53	6.29
Blood	7.34	7.26	7.26	7.28	7.30	7.29	7.28
EDTA-K_2	4.80	4.77	4.83	4.87	4.79	4.80	4.81
Sodium heparin	7.64	7.50	7.66	7.68	7.70	7.69	7.65
Sodium citrate	6.13	6.20	6.10	6.14	6.12	6.10	6.13
Blood (anticoagulated with EDTA-K_2)	7.23	7.25	7.30	7.28	7.22	7.23	7.25
Blood (anticoagulated with sodium heparin)	7.41	7.45	7.60	7.49	7.47	7..50	7.48
Blood (anticoagulated with sodium citrate)	6.63	6.74	6.5	6.67	6.7	6.61	6.64
